# Supplementary material for: Transcriptomics of MASLD Pathobiology in African American Patients in the Washington DC Area
Source: Int J Mol Sci. 2023 Nov 23;24(23):16654. doi: 10.3390/ijms242316654 (PMC10706626; doi:10.3390/ijms242316654)
Supplement: Supplementary file 1 [file ijms-24-16654-s001.zip › Supplementary Figures.pdf]

Supplementary Figures:

Supplementary Figure S1:

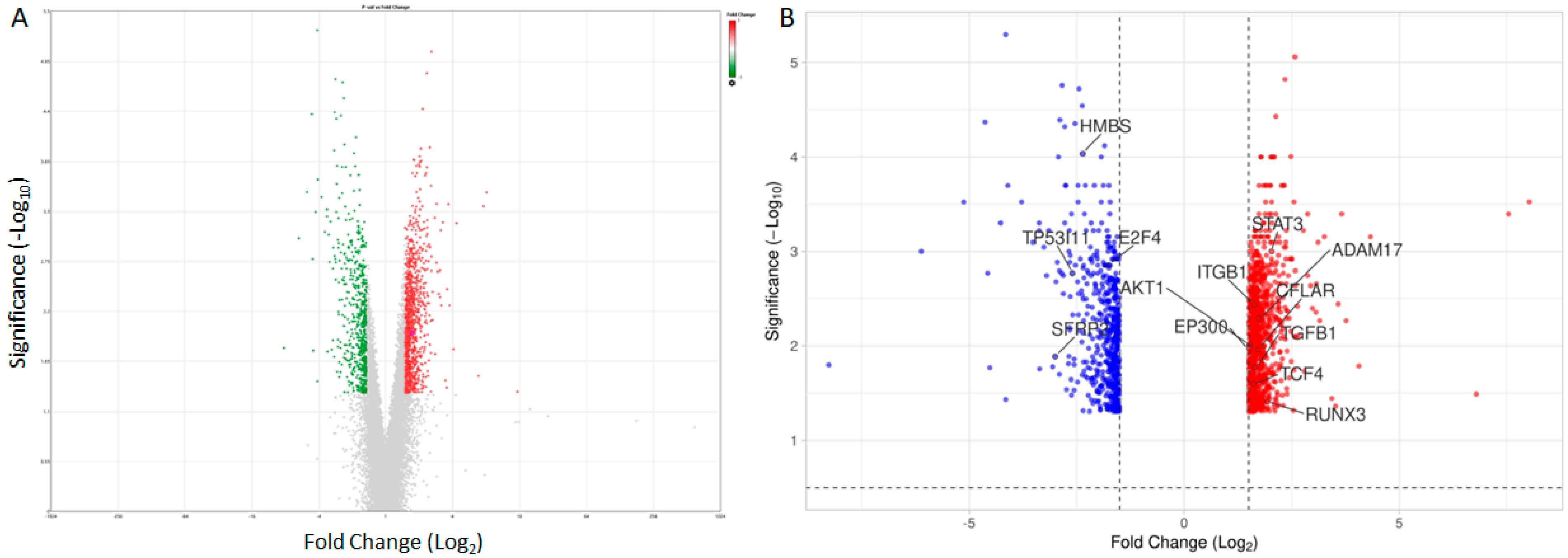

Supplementary Figure S1A: Volcano plot of the distribution of all differentially expressed genes captured by global expression (n=535; Filtered at Fold Change: +/-1.5′ p value <0.05), total 361 upregulated genes (red dots) and 174 downregulated genes (green dots). In a volcano plot, the most upregulated genes are towards the right, the most downregulated genes are towards the left, and the most statistically significant genes are towards the top. Figure S1B: Common genes observed by TLDA.

Supplementary Figure S2:

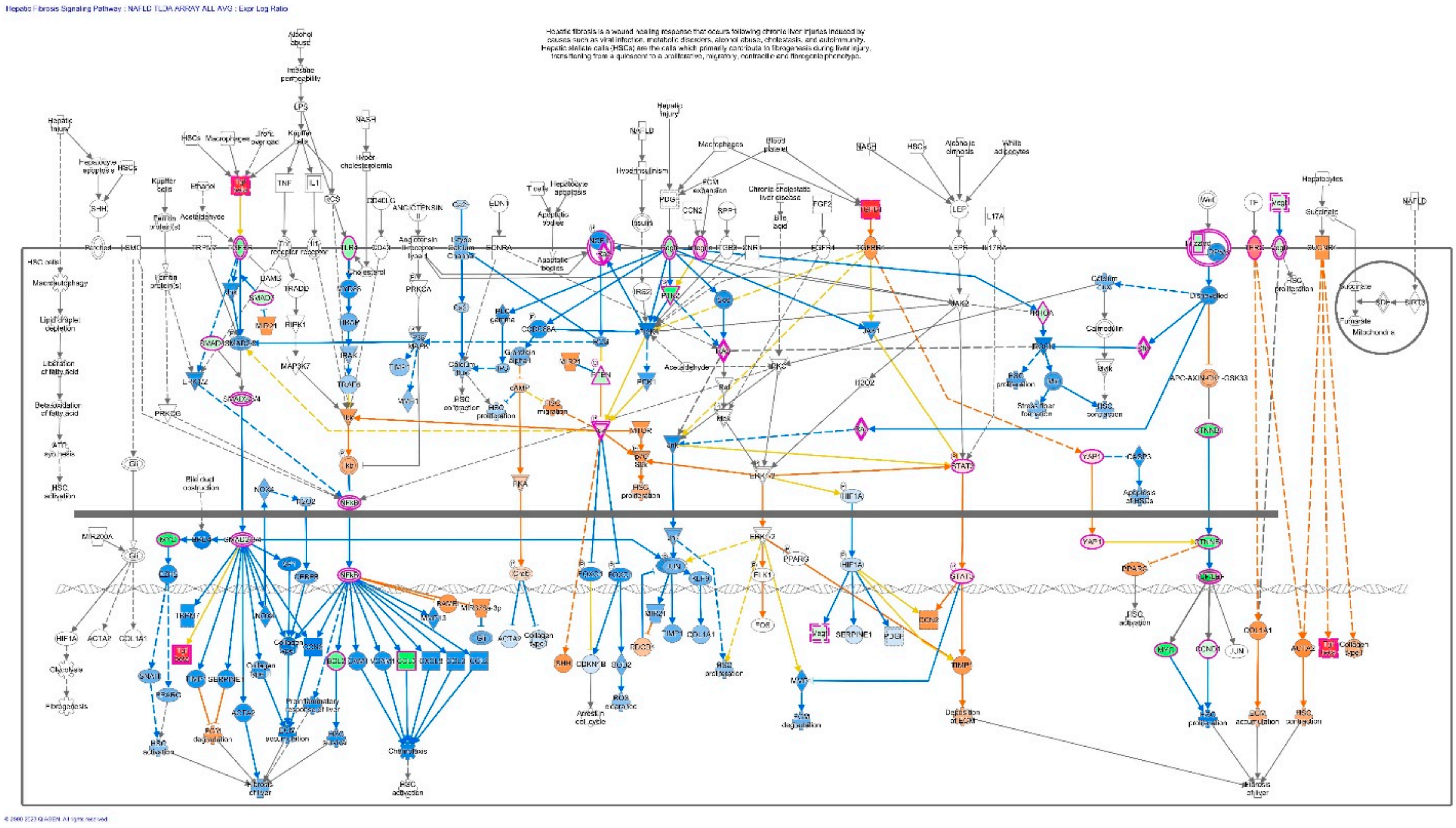

Supplementary Figure S2: Role of TGFB1 in Hepatic Fibrosis Signaling Pathway. The data was generated using Ingenuity Pathway Analysis (IPA) from QIAGEN, USA, March 2023 Release, and incorporated the relative gene expression levels measured in the patients' group.[47]
