# Supplementary material for: Transcriptomics of MASLD Pathobiology in African American Patients in the Washington DC Area
Source: Int J Mol Sci. 2023 Nov 23;24(23):16654. doi: 10.3390/ijms242316654 (PMC10706626; doi:10.3390/ijms242316654)
Supplement: Supplementary file 1 [file ijms-24-16654-s001.zip › Supplementary Tables.pdf]

Transcriptomics of MASLD Pathobiology in African American Patients in The Washington DC Area

Tanmoy Mondal et al.

Supplementary Table S1:

TaqMan® Array Plate; Target genes: 82; Controls: 14; Total assays: 96

	Assay ID	Gene Symbol(s)	Gene Name(s)
1	Hs99999901_s1	*18s rRNA*	-
2	Hs01041915_m1	*ADAM17*	ADAM metallopeptidase domain 17
3	Hs00178289_m1	*AKT1*	AKT serine/threonine kinase 1
4	Hs01048042_m1	*ANGPT2*	angiopoietin 2
5	Hs00180269_m1	*BAX*	BCL2 associated X, apoptosis regulator
6	Hs00608023_m1	*BCL2*	BCL2, apoptosis regulator
7	Hs00236329_m1	*BCL2L1*	BCL2 like 1
8	Hs00609632_m1	*BID*	BH3 interacting domain death agonist
9	Hs01112284_m1	*BIRC2*	baculoviral IAP repeat containing 2
10	Hs04194392_s1	*BIRC5*	baculoviral IAP repeat containing 5
11	Hs01018151_m1	*CASP8*	caspase 8
12	Hs00982282_m1	*CCL5*	C-C motif chemokine ligand 5
13	Hs00765553_m1	*CCND1*	cyclin D1
14	Hs00153380_m1	*CCND2*	cyclin D2
15	Hs01023894_m1	*CDH1*	cadherin 1
16	Hs01004530_m1	*CDH13*	cadherin 13
17	Hs00923894_m1	*CDKN2A*	cyclin dependent kinase inhibitor 2A
18	Hs00153439_m1	*CFLAR*	CASP8 and FADD like apoptosis regulator
19	Hs00355049_m1	*CTNNB1*	catenin beta 1
20	Hs00607978_s1	*CXCR4*	C-X-C motif chemokine receptor 4
21	Hs00368995_m1	*DAB2IP*	DAB2 interacting protein
22	Hs00183436_m1	*DLC1*	DLC1 Rho GTPase activating protein
23	Hs00153451_m1	*E2F1*	E2F transcription factor 1
24	Hs01099999_m1	*EGF*	epidermal growth factor
25	Hs01076078_m1	*EGFR*	epidermal growth factor receptor
26	Hs00914223_m1	*EP300*	E1A binding protein p300
27	Hs04187499_m1	*FADD*	Fas associated via death domain
28	Hs00236330_m1	*FAS*	Fas cell surface death receptor
29	Hs00179987_m1	*FHIT*	fragile histidine triad
30	Hs01052961_m1	*FLT1*	fms related tyrosine kinase 1
31	Hs00275833_s1	*FZD7*	frizzled class receptor 7

32	Hs04188837_g1	*GADD45B*	growth arrest and DNA damage inducible beta
33	Hs02512067_s1	*GSTP1*	glutathione S-transferase pi 1
34	Hs00300159_m1	*HGF*	hepatocyte growth factor
35	Hs01011015_m1	*HHIP*	hedgehog interacting protein
36	Hs00978050_g1	*HRAS*	HRas proto-oncogene, GTPase
37	Hs04188276_m1	*IGF2*	insulin like growth factor 2
38	Hs00236877_m1	*IGFBP1*	insulin like growth factor binding protein 1
39	Hs00365742_g1	*IGFBP3*	insulin like growth factor binding protein 3
40	Hs00178563_m1	*IRS1*	insulin receptor substrate 1
41	Hs00559595_m1	*ITGB1*	integrin subunit beta 1
42	Hs00911700_m1	*KDR*	kinase insert domain receptor
43	Hs01547250_m1	*LEF1*	lymphoid enhancer binding factor 1
44	Hs01050896_m1	*MCL1*	BCL2 family apoptosis regulator
45	Hs01565584_m1	*MET*	MET proto-oncogene, receptor tyrosine kinase
46	Hs00954125_m1	*MSH2*	mutS homolog 2
47	Hs00989003_m1	*MSH3*	mutS homolog 3
48	Hs00757841_m1	*MTDH*	metadherin
49	Hs00153408_m1	*MYC*	v-myc avian myelocytomatosis viral oncogene homolog
50	Hs00765730_m1	*NFKB1*	nuclear factor kappa B subunit 1
51	Hs00180035_m1	*NRAS*	neuroblastoma RAS viral oncogene homolog
52	Hs00170168_m1	*OPCML*	opioid binding protein/cell adhesion molecule like
53	Hs00998018_m1	*PDGFRA*	platelet derived growth factor receptor alpha
54	Hs01598309_m1	*PIN1*	peptidylprolyl cis/trans isomerase, NIMA-interacting 1
55	Hs02621230_s1	*PTEN*	phosphatase and tensin homolog
56	Hs00153133_m1	*PTGS2*	prostaglandin-endoperoxide synthase 2
57	Hs01056457_m1	*PTK2*	protein tyrosine kinase 2
58	Hs01547324_gH	*PYCARD*	PYD and CARD domain containing
59	Hs01902432_s1	*RAC1*	ras-related C3 botulinum toxin substrate 1 (rho family, small GTP binding protein Rac1)
60	Hs00200394_m1	*RASSF1*	Ras association domain family member 1
61	Hs01078066_m1	*RB1*	RB transcriptional corepressor 1
62	Hs01022646_m1	*RELN*	reelin
63	Hs00357608_m1	*RHOA*	ras homolog family member A
64	Hs00231709_m1	*RUNX3*	runt related transcription factor 3
65	Hs00293258_m1	*SFRP2*	secreted frizzled related protein 2
66	Hs00929647_m1	*SMAD4*	SMAD family member 4
67	Hs00998193_m1	*SMAD7*	SMAD family member 7
68	Hs00705164_s1	*SOCS1*	suppressor of cytokine signaling 1
69	Hs02330328_s1	*SOCS3*	suppressor of cytokine signaling 3

70	Hs00374280_m1	*STAT3*	signal transducer and activator of transcription 3
71	Hs00162613_m1	*TCF4*	transcription factor 4
72	Hs00972656_m1	*TERT*	telomerase reverse transcriptase
73	Hs00608187_m1	*TGFA*	transforming growth factor alpha
74	Hs00998133_m1	*TGFB1*	transforming growth factor beta 1
75	Hs00234253_m1	*TGFBR2*	transforming growth factor beta receptor 2
76	Hs00152939_m1	*TLR4*	toll like receptor 4
77	Hs00366278_m1	*TNFRSF10B*	TNF receptor superfamily member 10b
78	Hs00921974_m1	*TNFSF10*	tumor necrosis factor superfamily member 10
79	Hs01034249_m1	*TP53*	tumor protein p53
80	Hs00900055_m1	*VEGFA*	vascular endothelial growth factor A
81	Hs01103751_m1	*WT1*	Wilms tumor 1
82	Hs00745222_s1	*XIAP*	X-linked inhibitor of apoptosis
83	Hs00902712_g1	*YAP1*	Yes associated protein 1
84	Hs00608519_m1	*MRPL19*	mitochondrial ribosomal protein L19
85	Hs00237047_m1	*YWHAZ*	tyrosine 3-monooxygenase/tryptophan 5-monooxygenase activation protein zeta
86	Hs99999906_m1	*PGK1*	phosphoglycerate kinase 1
87	Hs00201226_m1	*CASC3*	cancer susceptibility candidate 3
88	Hs00609297_m1	*HMBS*	hydroxymethylbilane synthase
89	Hs00183533_m1	*IPO8*	importin 8
90	Hs00153277_m1	*CDKN1B*	cyclin dependent kinase inhibitor 1B
91	Hs00172187_m1	*POLR2A*	RNA polymerase II subunit A
92	Hs00245445_m1	*ABL1*	ABL proto-oncogene 1, non-receptor tyrosine kinase
93	Hs00362795_g1	*PES1*	pescadillo ribosomal biogenesis factor 1
94	Hs01102345_m1	*RPL37A*	ribosomal protein L37a
95	Hs99999910_m1	*TBP*	TATA-box binding protein
96	Hs99999911_m1	*TFRC*	transferrin receptor

Supplementary Table S2: Genes associated with liver disease significantly dysregulated in NAFLD subjects in this study.

Gene Name	Gene Name(s)	Expression Status
HMBS	Hydroxymethylbilane synthase	**Downregulated**
E2F1	E2F transcription factor 1	**Downregulated**
TP53	Tumor protein p53	**Downregulated**
SFRP2	Secreted frizzled related protein 2	**Downregulated**
STAT3	Signal transducer and activator of transcription 3	**Upregulated**
ITGB1	Integrin subunit beta 1	**Upregulated**
AKT1	AKT serine/threonine kinase 1	**Upregulated**
ADAM17	ADAM metallopeptidase domain 17	**Upregulated**
CFLAR	CASP8 and FADD like apoptosis regulator	**Upregulated**
TGFB1	Transforming growth factor beta 1	**Upregulated**
EP300	E1A binding protein p300	**Upregulated**
TCF4	Transcription factor 4	**Upregulated**
RUNX3	Runt related transcription factor 3	**Upregulated**

Supplementary Table S3: List of Focus Molecules observed in the top networks by global gene expression analysis. The table containing gene names and synonyms was extracted from Ingenuity Pathway Analysis (IPA), QIAGEN, Germany, March 2023 Release [21]. In this table, we have included the relative gene expression levels (experimental fold change) measured in the NAFLD patients compared to the control.

Symbol	Synonym(s)	Entrez Gene Name	Affymetrix	Expr Fold Change
ADGRE2	adhesion G protein-coupled receptor E2, CD312, CD97, EMR2, VBU	adhesion G protein-coupled receptor E2	TC1900009838.hg.1	2.79
ASPM	abnormal spindle microtubule assembly, ASP, assembly factor for spindle microtubules, CALMBP1, D330028K02Rik, FLJ10517, FLJ10517 FIS, FLJ12505 FIS, MCPH5, Sha1	assembly factor for spindle microtubules	TC0100016831.hg.1	-1.31
BMPR1A	10q23del, 1110037I22Rik, ACVRLK3, ALK-3, Bmpr, BMPR-IA, bone morphogenetic protein receptor type 1A, bone morphogenetic protein receptor, type 1A, CD292, JIP, SKR5	bone morphogenetic protein receptor type 1A	TC1000008315.hg.1	1.99
CEACAM 3	CD66D, CEA, cea12, Ceacam5, CEA cell adhesion molecule 3, Cear, CGM1, Cgm4, CGM4AA, EG384557, RATCEAA, RATCGM4AA, W264, W282	CEA cell adhesion molecule 3	TC1900008161.hg.1	1.83
CEACAM 5	1600029H12Rik, CD66e, CEA, CEA cell adhesion molecule 5, Psg30	CEA cell adhesion molecule 5	TC1900011741.hg.1	1.55
CENPE	C530022J18, centromere protein E, KIF10, MCPH13, PPP1R61	centromere protein E	TC0400011477.hg.1	-1.6
CKS2	1110038L14Rik, CDC28 protein KINASE REGULATORY subunit 2, CDC28 REGULATORY subunit 2, CKSHS2, P13SUC1, RGD1562047	CDC28 protein kinase regulatory subunit 2	TC0900007863.hg.1	-1.94
CSF2RB	AI848964, AIC2A, AIC2B, Bc, Beta C, betaGMR, beta IL-3, Bil3, CD131, CDw131, colony stimulating factor 2 receptor, beta 2, low-affinity (granulocyte-macrophage), colony stimulating factor 2 receptor, beta, low-affinity (granulocyte-	colony stimulating factor 2 receptor subunit beta	TC2200007273.hg.1	2.03

	macrophage), colony stimulating factor 2 receptor subunit beta, colony stimulating factor 2 receptor subunit β, colony stimulating factor 2 receptor, β 2, low-affinity (granulocyte-macrophage), colony stimulating factor 2 receptor, β, low-affinity (granulocyte-macrophage), Csf2r, Csf2rb1, Csf2rb2, Csfgmrb, Gm-csfr beta IT, Gm csf receptor β, Gmcsf receptor β c, GM-CSFR β, Gm-csfr β IT, Gmr β c, Granulocyte-macrophage colony stimulating factor 2 receptor, beta 1, Granulocyte-macrophage colony stimulating factor 2 receptor, β 1, Il3r, IL3RB, Il3rb1, Il3rb2, Il3rb common, IL3R beta 1, IL3R β 1, IL5RB, Il-5Rbetac, Il-5 receptor β chain, Il-5Rβc, SMDP5, β C, β chain of il 3 receptor, β IL-3			
CTSK	Cathepsin K, catK, CTS02, CTSO, CTSO1, CTSO2, MMS10-Q, Ms10q, PKND, PYCD	cathepsin K	TC0100015754.hg.1	1.91
DOCK4	5330406C03, 6330411N01RIK, AF263288, C030023J22, dedicator of cytokinesis 4, mKIAA0716	dedicator of cytokinesis 4	TC0700012276.hg.1	2.09
E2F4	2010111M04Rik, E2F transcription factor 4	E2F transcription factor 4	TC1600008149.hg.1	-1.88
EFHC1	1700029F22RIK, dJ304B14.2, EF-hand domain containing 1, EF-hand domain (C-terminal) containing 1, EJM1, LOC684521, mRib72-1, myoclonin1, POC9, RIB72	EF-hand domain containing 1	TC0600008255.hg.1	1.29
EGLN1	C1orf12, ECYT3, egl-9 family hypoxia-inducible factor 1, HALAH, Hif-p4h-2, HIF-PH2, HPH-2, ORF13, PHD-2, SM-20, ZMYND6	egl-9 family hypoxia inducible factor 1	TC0100017730.hg.1	1.62
GAB2	AI463667, D130058I17Rik, GRB2-associated binding protein 2, growth factor receptor bound protein 2-associated protein 2, p97, p98	GRB2 associated binding protein 2	TC1100011744.hg.1	2.32

HMGB3	high mobility group box 3, HMG-2a, HMG-4, MCOPS13, RGD1564407	high mobility group box 3	TC0X00008724.hg.1	-1.44
LYN	Hck-2, JTK8, LOC100862590, LYN KINASE, LYN proto-oncogene, Src family tyrosine kinase, p53/56 Lyn, p53Lyn, p53/p56 Lyn, p56Lyn	LYN proto-oncogene, Src family tyrosine kinase	TC0800007688.hg.1	1.3
MRTO4	2610012O22Rik, AC163633.1, C1orf33, dJ657E11.4, EG668455, Gm5633, Gm9178, Mg684, mRNA turnover 4, pseudogene 1, mRNA turnover 4, pseudogene 2, mRNA turnover 4, ribosome maturation factor, MRT4, MRT4 homolog, ribosome maturation factor, Mrto4-ps1, Mrto4-ps2, RGD1311709	MRT4 homolog, ribosome maturation factor	TC0100007160.hg.1	-1.58
MYBL2	B-MYB, MYBB, MYB proto-oncogene like 2, MYB-Related Protein B, myeloblastosis oncogene-like 2	MYB proto-oncogene like 2	TC2000007438.hg.1	-1.4
PIWIL4	9230101H05, 9230101H05Rik, HIWI2, mAgo5, MIWI2, PIWI4, piwi-like RNA-mediated gene silencing 4	piwi like RNA-mediated gene silencing 4	TC1100013082.hg.1	1.27
POLA1	alpha PRIMASE p180, DNA polymerase alpha, DNA polymerase alpha 1, catalytic subunit, DNA polymerase alpha subunit 1, DNA polymerase α, DNA polymerase α 1, catalytic subunit, DNA polymerase α subunit 1, NSX, p180, POLA, POLYMERASE alpha, polymerase (DNA directed), alpha 1, polymerase (DNA directed), α 1, POLYMERASE α, VEODS, α PRIMASE p180	DNA polymerase alpha 1, catalytic subunit	TC0X00006823.hg.1	-1.53
PTX3	LOC682447, pentraxin 3, pentraxin related gene, TNFAIP5, TSG-14	pentraxin 3	TC0300009301.hg.1	-1.87
SFTPC	BRICD6, pro-SpC, PSP-C, SFTP2, SMDP2, SP5, SP-C, surfactant associated protein C, surfactant protein C	surfactant protein C	TC0800012277.hg.1	-1.3

SRRM2	5033413A03Rik, AA410130, Cwc21, CWF21, HSPC075, KIAA0324, mKIAA0324, serine/arginine repetitive matrix 2, SRL300, SRm300	serine/arginine repetitive matrix 2	TC1600006633.hg.1	1.3
SSX2IP	ADIP, AU042321, hMsd1, LOC100909794, LOC308023, SSX family member 2 interacting protein, synovial sarcoma, X 2 interacting protein	SSX family member 2 interacting protein	TC0100014762.hg.1	-1.71
TFDP2	1110029I05RIK, A330080J22RIK, DP2, DP-3, Tcfdp2, transcription factor Dp-2	transcription factor Dp-2	TC0300012654.hg.1	-2.91
TGFB1	Beta Ig-h3, CED, DPD1, IBDIMDE, LAP, TGFB, TGFbeta, TGF-beta1, TGF-β, TGF-β1, tgf-β(1), transforming growth factor beta 1, transforming growth factor, beta 1, transforming growth factor-β 1, Transforming growth factor-β(1), transforming growth factor, β 1, β Ig-h3	transforming growth factor beta 1	TC1900010743.hg.1	1.64
TPM1	AI854628, alpha-TM, BI546321, BQ310475, C15ORF13, C76867, CMD1Y, CMH3, HEL-S-265, HTM-alpha, HTM-α, LVNC9, TM1, Tm3, Tma2, Tm alpha, Tmpa, TMSA, Tm α, TPM1kappa, TROPOMYOSIN 1, α-TM	tropomyosin 1	TC1500007510.hg.1	-1.82
TTK	CT96, ESK, Esk1, MPH1, MPS1, MPS1L1, PYT, TTK protein kinase	TTK protein kinase	TC0600008630.hg.1	1.61
UMPS	1700095D23RIK, BB164745, OPRT, Orotidylic acid (OMP) decarboxylase, uridine monophosphate synthetase	uridine monophosphate synthetase	TC0300008604.hg.1	-1.41
ZFP36	G0S24, GOS24, GROWTH FACTOR INDUCIBLE NUCLEAR protein 475, NUP475, RNF162A, TIS11, TIS11D, TISII, TRISTETRAPROLIN, TRISTETRAPROLINE, TTP, ZFP36 ring finger protein, zinc finger protein 36, ZINC FINGER TRANSCRIPTIONAL REGULATOR	ZFP36 ring finger protein	TC1900008057.hg.1	1.59
ZNF652	9530033F24Rik, B130006D01Rik, RGD1566329, Zfp652, zinc finger protein 652	zinc finger protein 652	TC1700011038.hg.1	1.99

Supplementary Table S4: List of Focus Molecules observed in the top networks by TLDA gene expression analysis. The information the table including gene names and synonyms were exported from Ingenuity Pathway Analysis (IPA), QIAGEN, Germany, March 2023 Release [21]. We have incorporated the relative gene expression (experimental fold change) levels measured in the patients' group.

Symbol	Synonym(s)	Entrez Gene Name	Life Technologies (Applied Biosystems)	Expr Log Ratio
AKT1	AKT, AKT serine/threonine kinase 1, LTR-akt, PKB, PKB/Akt, PKB-ALPHA, PKB-α, PRKBA, Protein kinase B, RAC, RAC-ALPHA, RAC-α, Thymoma proto-oncogene 1, thymoma viral proto-oncogene 1	AKT serine/threonine kinase 1	Hs00178289_m1	0.040885
CCL5	C-C motif chemokine ligand 5, chemokine (C-C motif) ligand 5, D17S136E, eoCP, MuRantes, RANTES, RNTES, SCYA5, Similar to merantes, SISd, SIS-delta, SIS-δ, TCP228	C-C motif chemokine ligand 5	Hs00982282_m1	-0.22712
CXCR4	b2b220Clo, CD184, CHEMOKINE CXC4 receptor, chemokine (C-X-C motif) receptor 4, CHEMOKINE receptor 4, Cmkar4, C-X-C motif chemokine receptor 4, D2S201E, FB22, FUSIN, HM89, HSY3RR, LAP-3, LCR1, LESTR, LOC100047410, NPY3R, NPYR, NPYRL, NPYY3R, PB-CKR, PBSF/SDF-1, Sdf1r, WHIM, WHIMS, WHIMS1	C-X-C motif chemokine receptor 4	Hs00607978_s1	-0.23092
EGF	beta-urogastrone, CN02, EGF-1, epidermal growth factor, epidermal growth factors, epidermal growth factor-urogastrone, HOMG4, LOC103691699, Rac1/Cdc42 activator II, URG, urogastrone, β-urogastrone	epidermal growth factor	Hs01099999_m1	-0.15854
FLT1	FLT, FMS-like tyrosine kinase 1, fms related receptor tyrosine kinase 1, VEGFR-1	fms related receptor tyrosine kinase 1	Hs01052961_m1	0.000316
HGF	C230052L06Rik, DFNB39, F-TCF, hepatocyte growth factor, HGFB, HGF/SF, HPTA, NK1, NK2, SF, SF/HGF	hepatocyte growth factor	Hs00300159_m1	-0.22871

HRAS	C-BAS/HAS, C-HA-RAS, C-HA-RAS1, C-H-RAS, c-rasHa, CTLO, HAMSV, HA-RAS, Harvey-ras, Harvey rat sarcoma virus oncogene, HRAS1, H-RASIDX, HRas proto-oncogene, GTPase, Kras2, RAS, RASH1, RAS HA, V-H-RAS	HRas proto-oncogene, GTPase	Hs00978050_g1	0.116924
IGFBP3	BP-53, IBP3, IGBP3, IGF binding protein 3, IGFI BP3, IGgfbp3, Insulin-like growth factor-binding protein 3	insulin like growth factor binding protein 3	Hs00365742_g1	0.110458
ITGB1	4633401G24RIK, beta1-Integrin, CD29, CD29 antigen, FNRB, Gm9863, GPIIA, integrin beta 1 (fibronectin receptor beta), integrin subunit beta 1, integrin subunit β 1, Integrin β 1, integrin β 1 (fibronectin receptor β), Integrin β 1 receptor, LOC102556297, MDF2, MSK12, VLAB, VLA-BETA, VLA-β, β1-Integrin, β(1)-integrins	integrin subunit beta 1	Hs00559595_m1	0.035153
MET	AI838057, AUTS9, c-Met, DA11, DFNB97, HGF, HGF Binding, HGFR, LOC360378, met proto-oncogene, MET proto-oncogene, receptor tyrosine kinase, MetR, PAR4, RCCP2	MET proto-oncogene, receptor tyrosine kinase	Hs01565584_m1	0.079116
PDGFRA	APDGFR, CD140A, LOC102554024, PDGFACE, Pdgfar, PDGFR-2, PDGFR alpha, Pdgf receptor-α, PDGFR α, PDGF α R, Pdgf α Receptor, platelet derived growth factor receptor alpha, platelet derived growth factor receptor, alpha polypeptide, platelet derived growth factor receptor α, platelet derived growth factor receptor, α polypeptide, α-pdgfr	platelet derived growth factor receptor alpha	Hs00998018_m1	-0.11542
PTEN	10q23del, 2310035O07RIK, A130070J02Rik, B430203M17RIK, BZS, CWS1, DEC, GLM2, MHAM, Mmac, MMAC1, MUTATED IN MULTIPLE ADVANCED CANCERS, mutated in multiple advanced cancers 1, O54857, phosphatase and tensin homolog, PTEN1, PTENbeta, TEP1	phosphatase and tensin homolog	Hs02621230_s1	-0.07493
PTGS2	Cox, COX-2, CYCLO-OXYGENASE 2, GRIPGHS, hCox-2, hnCOX-2, INDUCIBLE CYCLOOXYGENASE, PES-2, PGG/HS, PGHS-2, PGH synthase 2,	prostaglandin-endoperoxide synthase 2	Hs00153133_m1	-0.15236

	Pgi2 synthase, Pgs2, Pgsi, PHS-2, PHS II, Prostaglandin endoperoxide synthase 2, TIS10			
PTK2	FADK, FADK 1, FAK, FAK1, FAK related non-kinase, p125FAK, pp125FAK, PPP1R71, protein tyrosine kinase 2, PTK2 protein tyrosine kinase 2, TYROSINE KINASE 2	protein tyrosine kinase 2	Hs01056457_m1	-0.1887
PYCARD	9130417A21Rik, ASC, CARD5, hASC, masc, MGC10332, PYD and CARD domain containing, TMS, TMS-1, TNS1	PYD and CARD domain containing	Hs01547324_gH	-0.1119
RAC1	AL023026, D5Ertd559e, MIG5, MRD48, p21-RAC, p21-Rac1, Rac, Rac family small GTPase 1, TC-25	Rac family small GTPase 1	Hs01902432_s1	-0.07613
SMAD7	CRCS3, MADH7, MADH8, SMAD family member 7	SMAD family member 7	Hs00998193_m1	-0.07433
SOCS3	ATOD4, CIS3, CISH3, EF-10, Soc3, Sosc3, SSI-3, Suppressor of cytokine signaling 3	suppressor of cytokine signaling 3	Hs02330328_s1	-0.00201
TGFA	ETGF, RATTGFAA, TFGA, TGFAA, Tgf alpha, Tgf α, transforming growth factor alpha, transforming growth factor-α, wa-1	transforming growth factor alpha	Hs00608187_m1	-0.00053
TGFB1	Beta Ig-h3, CED, DPD1, IBDIMDE, LAP, TGFB, TGFbeta, TGF-beta1, TGF-β, TGF-β1, tgf-β(1), transforming growth factor beta 1, transforming growth factor, beta 1, transforming growth factor-β 1, Transforming growth factor-β(1), transforming growth factor, β 1, β Ig-h3	transforming growth factor beta 1	Hs00998133_m1	0.353578
TLR4	ARMD10, CD284, HTOLL, Ly87, Ran/M1, Rasl2-8, Tlrp4, TOLL, toll-like receptor 4, TOLL receptor	toll like receptor 4	Hs00152939_m1	-0.11595
VEGFA	Gd-vegf, MVCD1, vascular endothelial growth factor A, VEGF, VEGF111, Vegf-3, VPF	vascular endothelial growth factor A	Hs00900055_m1	-0.03679

Supplementary Table S5: NAFLD Participants with detailed FibroScan and Dyslipidemia data.

Participant Sl No.	Participant Type	Gender	FibroScan	Dyslipidemia (LDL|HDL|Tri)
1	NAFLD	Female	F0, S3	-
2	NAFLD	Female	-	136|37|217
3	NAFLD	Male	F0, S0	100|51|87
4	NAFLD	Female	F2, S2	185|64|202
5	NAFLD	Female	F0-F1, S2	117|65|105
6	NAFLD	Female	F0, S2-S3	157|55|95
7	NAFLD	Male	F2, S1-S2	65|60|103
8	NAFLD	Male	F0, S0	67|31|311
9	NAFLD	Male	F4, S1-S2	18|24|59
10	NAFLD	Female	F0, S2-S3	63|63|61
11	NAFLD	Female	-	-
12	NAFLD	Male	F0-F1, S3	36|21|306
13	NAFLD	Female	F2-F3, S3	107|72|69
14	NAFLD	Male	F0-F1, S1	148|38|81
15	NAFLD	Female	-	114|39|163
16	NAFLD	Female	F0-F1, S0	107|68|64
17	NFALD	Male	F0-F1, S3	157|41|222
18	NFALD	Male	F0-F1, S0	160|38|150
19	NAFLD	Male	F0, S0	71|36|73
20	NAFLD	Male	F3, S0	119|76|67
21	NAFLD	Female	F3, S2-S3	-
22	NAFLD	Male	F2, S3	58|27|222
23	NAFLD	Female	F2, S3	93|48|127

*F0-Normal, F1-Mild, F3-Moderate, and F4-Severe.
**S0-Less than 11% (normal), S1-11% to 33%, S2-34% to 66%, S3-Greater than 67%.
*** To select the participants, we kept importance at BMI, HbA1c value, dyslipidemia, and presence of steatosis (as measured by FibroScan). For the purposes of liver steatosis, we considered the S grade on the FibroScan (S0 - S3). The higher the grade, the higher percentage of liver affected by fatty change.
